# Cortical Actin Nanodynamics Determines Nitric Oxide Release in Vascular Endothelium

**DOI:** 10.1371/journal.pone.0041520

**Published:** 2012-07-23

**Authors:** Johannes Fels, Pia Jeggle, Kristina Kusche-Vihrog, Hans Oberleithner

**Affiliations:** Institute of Physiology II, University of Muenster, Muenster, Germany; Center for Cancer Research, National Cancer Institute, United States of America

## Abstract

The release of the main vasodilator nitric oxide (NO) by the endothelial NO synthase (eNOS) is a hallmark of endothelial function. We aim at elucidating the underlying mechanism how eNOS activity depends on cortical stiffness (К_cortex_) of living endothelial cells. It is hypothesized that cortical actin dynamics determines К_cortex_ and directly influences eNOS activity. By combined atomic force microscopy and fluorescence imaging we generated mechanical and optical sections of single living cells. This approach allows the discrimination between К_cortex_ and bulk cell stiffness (К_bulk_) and, additionally, the simultaneous analysis of submembranous actin web dynamics. We show that К_cortex_ softens when cortical F-actin depolymerizes and that this shift from a gel-like stiff cortex to a soft G-actin rich layer, triggers the stiffness-sensitive eNOS activity. The results implicate that stiffness changes in the ∼100 nm phase of the submembranous actin web, without affecting К_bulk_, regulate NO release and thus determines endothelial function.

## Introduction

Endothelial cells form the inner layer of blood vessels and play an important role in the regulation of blood pressure and tissue perfusion, for they release vasoactive substances. Most important among these is the gaseous messenger nitric oxide (NO) [1]. In the healthy blood vessel, NO is synthesized by the endothelial NO synthase (eNOS) which is located in close vicinity to the plasma membrane [2]. Upon its release by the endothelial cells, NO induces a dilation of vascular smooth muscle cells which results in an increased vessel diameter, increased local tissue perfusion and decreased systemic blood pressure. Disturbance of eNOS activity, and hence NO release, may lead to severe endothelial dysfunction which is a predisposition of vascular diseases [3]. Recent studies have shown that the nanomechanical properties of endothelial cells control eNOS activity [4], [5], [6]. Especially the elasticity of the 50–100 nm thick sub-membranous layer, i.e. the cell cortex [7], correlates directly with endothelial NO release in an inverse manner [8]. A decreased stiffness of the cell cortex (cortical stiffness, К_cortex_) induces an increase in eNOS activity whereas an augmentation of К_cortex_ has the opposite effect [8]. Interestingly, this stiffness-dependent effect is independent of the elasticity of the cell centre (>300 nm below the plasma membrane), known as bulk stiffness (К_bulk_). Certain stimuli, e.g. extracellular potassium, influence К_cortex_ and hence improve NO release, but do not affect К_bulk_ [5]. This implies that the cell cortex represents a functional and independent compartment within the cell. The dependence of eNOS activity from К_cortex_ led to the postulation of the stiff endothelial cell syndrome (SECS) [9]. This mechanical property is expected to be responsible for the endothelial dysfunction leading to arterial hypertension and atherosclerosis. Several factors (e.g. plasma sodium, aldosterone) involved in these pathologies induce cortical stiffening followed by a decrease in eNOS activity [4], [10]. Although it is known that К_cortex_ directly correlates to eNOS activity, the determinant of К_cortex_ as well as the underlying mechanism is yet unknown.

In searching for the missing link between К_cortex_ and eNOS activity, some previous findings should be considered. Firstly, eNOS is located in the cell cortex at the inner face of the endothelial plasma membrane, at nanometre distance to the dynamic meshwork of actin filaments (F-actin) which forms the cortical cytoskeleton [2]. Secondly, eNOS associates with actin. An association with globular actin (G-actin) shows an increased activity as compared to an association with F-actin [11]. Thirdly, actin web dynamics directly correlates with cellular nanomechanics [12]. This is supported by finding that a depolymerisation of F-actin softens the cell [13], [14], [15], [16]. However, in these studies rather high concentrations of the respective actin-depolymerizing agents were used and an analysis focusing specifically on the cell cortex physiology is lacking. Combining these three findings, the functional state of the cortical actin web most probably determines К_cortex_ and thereby eNOS activity.

So far, a direct proof of the relationship between cortical actin, stiffness and eNOS activity is missing. This is due to a lack of proper techniques to simultaneously measure К_cortex_ and the cortical actin dynamics in living endothelial cells. The techniques used before to visualize the actin cytoskeleton involved labelling of actin by phalloidin-based probes (or the like) or the transfection of cells with DNA coding for actin-GFP fusion proteins. However, phalloidin is cytotoxic [17] and, similar to actin-GFP fusion proteins, interferes with cellular functions [18]. In addition, most experimental protocols require sample fixation and thus exclude the analysis of any dynamic intracellular processes in living cells. A promising marker to visualize actin dynamics is the recently introduced Lifeact-eGFP. This fluorescent marker labels F-actin in living cells without affecting the actin expression or its polymerization rate [19]. Therefore we developed a new method which proves the physiological relevance of stiffness-modulated eNOS activity via changes in the cortical F-actin/G-actin ratio. For this, we used a hybrid setup of an atomic force microscope (AFM) integrated into an epifluorescence microscope [20], [8]. The AFM enables mechanical tomography of the cell body, i.e. to distinguish precisely between the stiffness of the cell cortex and the bulk phase with sensitivity in the range of pico-Newton [13], [5]. Quantitative changes in the cortical actin web were simultaneously detected by epifluorescence microscopy using Lifeact-eGFP transfected endothelial cells. Optical resolution of the cell cortex was achieved by the analysis of the fluorescence intensity in the cell periphery. Additionally we analysed G-actin dependent eNOS activity by inhibiting the interaction of eNOS and actin using the inhibitor peptide P326TAT [11].

Using the described methods, we show that К_cortex_ is defined by cortical actin web dynamics. A cortical depolymerisation, and thereby a shift from F- to G-actin, is associated with a reduced К_cortex_. The higher G-actin content of the cell cortex leads to an increased interaction of eNOS and G-actin and thereby to an elevated NO release.

## Methods

### Solutions and Reagents

All reagents were purchased from Sigma-Aldrich (Steinheim, Germany) if not mentioned otherwise. During experiments the cells were continuously perfused with HEPES buffered solutions (in mM: 140 NaCl, 5 KCl, 1 MgCl_2_, 1 CaCl_2_, 5 glucose and 1 L-arginine adjusted to pH 7.4 at RT). CD buffer was supplemented with 50 nM cytochalasin D (1 mM stock in 100% ethanol). High extracellular K^+^ was achieved by isoosmotically increasing KCl from 5 to 12 mM. In some experiments 140 mM NaCl was replaced by sodium gluconate (140 mM) to reduce chloride concentration. All control buffers were supplemented with the respective amount of solvent or were isoosmotically adjusted with mannitol if required.

### Cell Culture

Bovine aortic endothelial cells (GM7373) [21] were cultured as described previously [4]. In brief, cells were cultured in minimal essential medium supplemented with 1% non-essential amino acids, 1% MEM vitamins (Invitrogen Corp., Karlsruhe, Germany) and 20% fetal bovine serum (FCS, PAA Clone, Coelbe, Germany). T_25_ culture flasks were used to culture the cells in an incubator at 37°C, 5% CO_2_ and 100% humidity.

### Lifeact-eGFP Transfection

**Figure 1:**
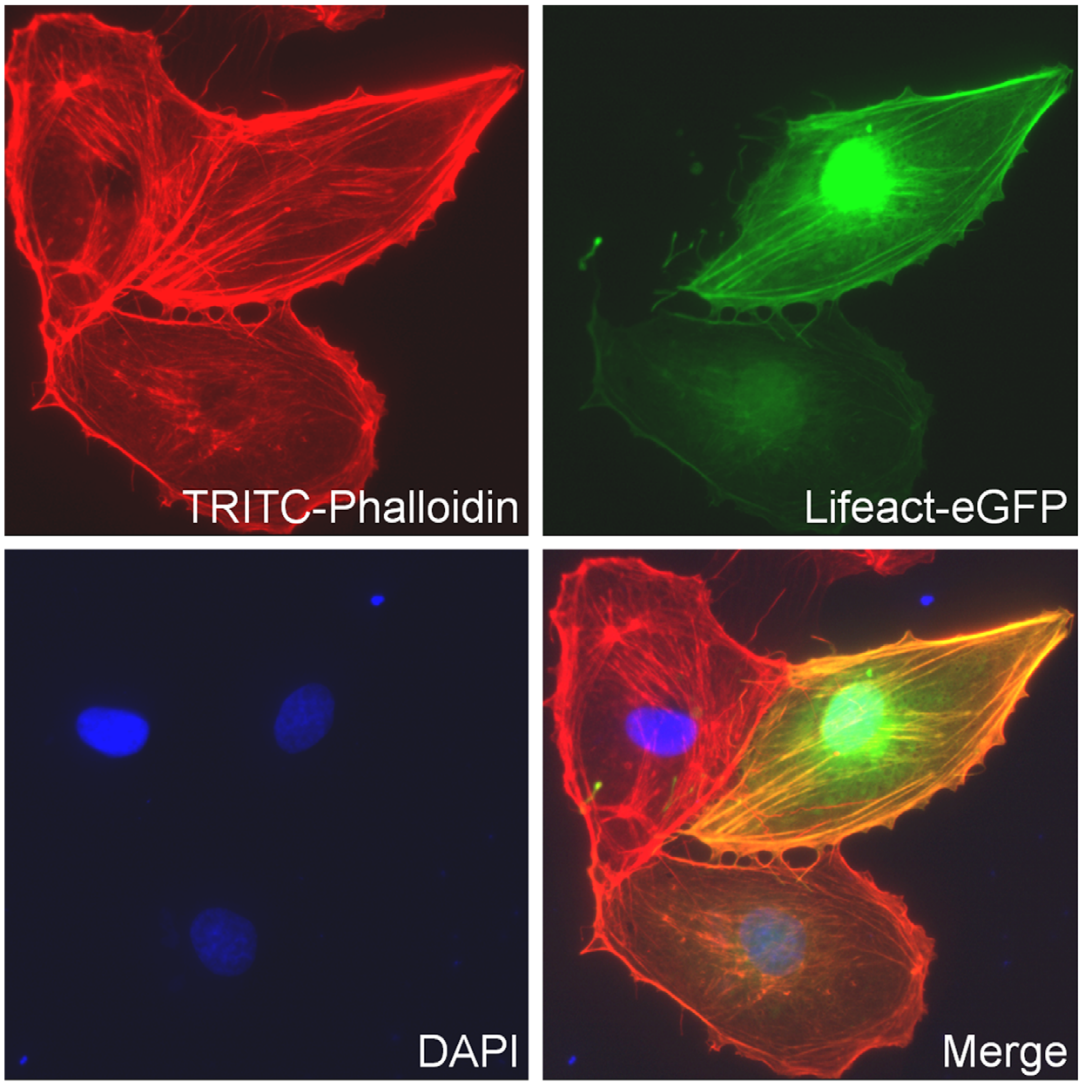
Lifeact verification. Double staining of F-actin in GM7373 bovine aortic endothelial cells using Lifeact-eGFP and TRITC-phalloidin. DAPI is used to stain nuclei. The merge image verifies that Lifeact-eGFP and TRITC-phalloidin are equally applicable to detect F-actin in endothelial cells.

GM7373 cells were transfected with Lifeact-eGFP to trace F-actin dynamics in living cells. Lifeact consists of a 17 amino acids long actin binding motif, derived from the *saccharomyces cerevisiae* protein Abp140 [22]. The peptide sequence has been ligated to the sequence of the enhanced green fluorescent protein eGFP, which allows tracing actin dynamics in living cells without interference of other signalling pathways or modification of actin expression and polymerization [19]. Cells used for measurements of Lifeact-eGFP fluorescence were cultured as described above. Three days prior to experiments, the cells were trypsinized, centrifuged and resuspended in OptiMEM medium (Invitrogen) supplemented with 10% FCS. The transfection reagent, using FuGENE6 transfection reagent (Roche, Mannheim, Germany) was prepared as described in the manufacturers protocol. In brief, 230 µl FCS-free OptiMEM Medium was mixed with 10 µl FuGENE6 Transfection Reagent and incubated for 10 minutes at room temperature (RT). Then, 5 µg Lifeact-eGFP DNA (in pEGFP-N1 vector, kindly provided by Dr. Roland Wedlich-Soldner, Max Planck Institute of Biochemistry, Martinsried, Germany) was added and the solution was gently mixed. After a second period of incubation (10 minutes at RT), the cells were added, mixed gently and then seeded on Ø 40 mm glass bottom dishes (WillCo Wells B.V., Amsterdam, Netherlands). To improve the transfection efficiency, the cells were subsequently incubated on a shaker (180 rpm) for another 40 minutes in the cell culture incubator. At the day of experiment, the cell monolayer was 100% confluent. To verify the specificity of Lifeact to label F-actin, GM7373 cells were stained with phalloidin-tetramethylrhodamine B isothiocyanate (phalloidin-TRITC). For this, transfected Lifeact-eGFP cells were fixed with 4% formaldehyde in phosphate buffered solution (PBS) for 15 minutes at RT. Subsequently, the cells were washed with PBS and incubated (30 min, RT) in 10% goat-normal-serum supplemented with phalloidin-TRITC (final 5 units/ml) and 4′,6-diamidino-2-phenylindole (DAPI, Invitrogen, final 0.1 µg/ml). Figure 1 shows a fluorescence image of a phalloidin-stained Lifeact-eGFP-expressing endothelial cell. The overlay of the Lifeact-eGFP and phalloidin-TRITC image verifies that both dyes match and approves Lifeact as a tool to visualize F-actin in endothelial cells.

### Simultaneous Measurements of Cortical Stiffness and Fluorescence Intensity

**Figure 2:**
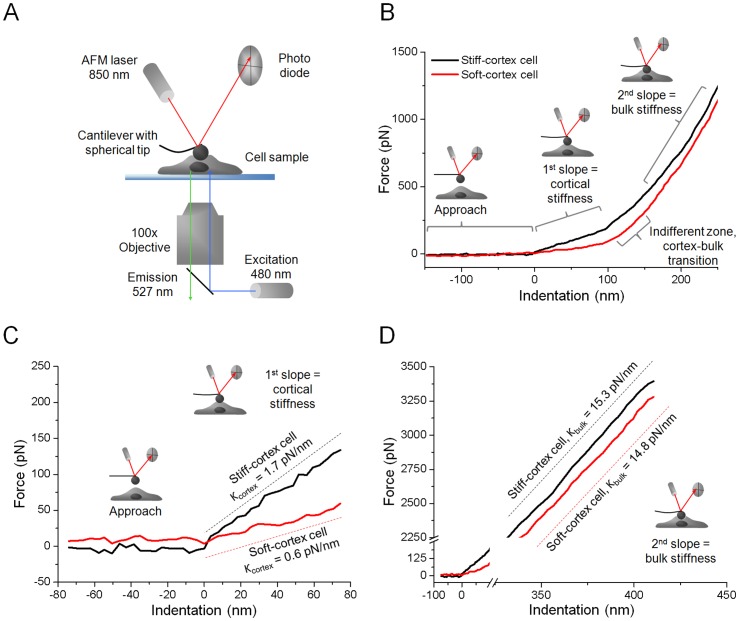
Stiffness measurements. a) Combined setup of an atomic force microscope integrated into an inverse epifluorescence microscope (mod. after [20]). b) Two force-indentation curves acquired on a single endothelial cell before (black) and after (red) cortical softening, induced by 50 nM cytochalasin D. Cells with a soft cortex may exhibit an indifferent zone of nonlinear stiffness in the area of the cortex-bulk-transition. c) Zoom-in of the approach and cortical indentation part of the force-indentation curves. Both curves are linear (no force needed) when the colloidal tip is approaching the cell. Indentation forces differ in soft-cortex and stiff-cortex cells. d) Force-indentation curves corresponding to the bulk phase of the cell. The curves show that bulk stiffness is unaffected by changes in the nanomechanics of the cell cortex.

AFM-based analysis of living cells allows the assessment of cell topography [23] as well as the analysis of protein interaction and stoichiometry [24], [25], [26]. Additionally, the AFM is the tool of choice to analyse regional stiffness of living cells [27]. A hybrid setup consisting of an AFM integrated into an epifluorescence microscope allows the simultaneous measurements of К_cortex_ and the actin dynamics of the cell cortex (Figure 2a). The Bioscope Catalyst AFM (Bruker, Karlsruhe, Germany) was used to perform К_cortex_ and К_bulk_ measurements ( = force tomography) as described previously [28], [29]. In brief, AFM-cantilevers (MLCT contact microlever, Bruker, spring constant approx. 0.01 pN/nm), equipped with 10 Ø µm colloidal tips (Novascan, Ames, IA, USA, for high resolution SEM image see [5]), were used to periodically indent the cells. A laser beam reflected from the backside of the cantilever is detected by a photo diode and allowing measurements of cantilever deflection. The large size of the spherical cantilever tip allowed measurements at very low forces (range: 200 pN) and thereby does not disturb cell function. By the determination of the spring constant as well as by measuring the deflection sensitivity of the cantilever, the force acting on the cantilever (an in turn the force exerted by the cantilever to the sample) can be calculated:
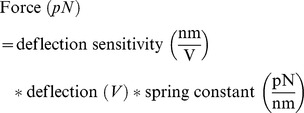



Additionally, by taking the piezo displacement into account, the indentation (deformation) of the sample can be determined:
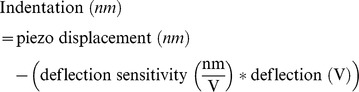



Subsequently, force-indentation-curves (F/I-curve), generated by plotting the force against the indentation, can be used to analyse the stiffness of different cell regions (cell depths). The stiffness (К) is an indicator of the mechanical resistance of a sample against a defined deformation (i.e. indentation). If the slope of the F/I-curve correlates to a linear fit, К can be estimated by:

Figure 2b displays two typical F/I-curves, the first one taken from a cell with a stiff cortex, the second one with a soft cortex. During the vertical approach, the cantilever does not bend since no force acts on the cantilever. Therefore, the stiffness of the cell (slope of both curves) tends to be ∼0 pN/nm (Figure 2b). As soon as the cantilever gets into contact to the cell surface and starts to indent the cell, force is exerted on the cantilever which results in cantilever bending. Hence, the slope of the F/I-curves changes to positive values. Interestingly, a typical F/I-curve shows a linear slope within the first 50 to 100 nm. This implicates the linear fit model to be appropriate for stiffness analysis (Figure 2c). A further indentation induces a shift in the F/I-curve, which shows again an almost linear behaviour. The second linear slope of the F/I-curves corresponds to the stiffness of the bulk phase of the cell (Figure 2d). It should be mentioned that the transition from the cortex to the bulk phase is not in any event characterized by a distinct shift of the slope of F/I-curves. As shown in Figure 2b, cells exhibiting a very soft cell cortex may show non-linear stiffness behaviour in the region of the cortex-bulk-transition. However, the cortex itself as well as the bulk region generally shows a F/I-curve with linear characteristics (R^2^≥0.96). Hence, to detect cortical stiffness, the first linear 50 nm of indentation were analysed whereas bulk stiffness was analysed in the linear region of 350–500 nm of indentation.

To verify that the first event of indentation definitely corresponds to the cell cortex (and not to e.g. membrane ruffles, glycocalyx or microvilli), we performed high resolution imaging of the cell surface. As shown in figure S1a, confluent GM7373 cells were fixed with 0.5% glutaraldehyde and imaged with MLCT cantilever in fluid (Bruker, spring constant 0.01 pN/nm). The magnified regions displayed in suppl. figure S1b show that the surface of endothelial cells is rather smooth and holds no deep ruffles or microvilli. Instead, the submembraneous actin filaments (arrowheads) as well as tiny invaginations (arrows), possibly corresponding to caveolae, can be imaged. Even an initial detection of the endothelial glycocalyx can be excluded since much lower forces and ramp parameters are necessary to detect the endothelial surface layer. Hence it is evident that the detected first slope of the F/I-curve matches to the cell cortex. To prevent mechanical alteration of the cell, a low indentation velocity of the cantilever (max. 1 µm/second) was chosen for experiments. Force-distance curves were acquired using the Nanoscope v7.30 software (Bruker) and data analysis was performed with Protein Unfolding and Nano-Indentation Software (PUNIAS, http://punias.voila.net/).

Simultaneously to the AFM measurements, the integrated DMI 6000 B inverted epifluorescence microscope (Leica, Microsystems, Wetzlar, Germany) was used for detection of Lifeact-eGFP fluorescence intensity. Epifluorescence is a eligible method since other fluorescence techniques do have lower time resolutions (e.g. confocal microscopy) or do not allow an illumination of the apical surface during constant perfusion (e.g. TIRF). The fluorophore was excited at 480/40 nm and emission was detected at 527/30 nm (Figure 2a). To obtain a high resolution, a 100× oil immersion objective was used for acquisition with an exposure time of 200 ms. Several regions of interest were selected in the peripheral regions of the transfected cells. Background fluorescence was subtracted as appropriate. The frequency of image (and force-distance curve) acquisition was set to 20 seconds in order to prevent photo bleaching. Fluorescence imaging and data analysis was performed using LAS AS 1.7.0 software (Leica). All experiments were performed at room temperature. Stiffness and fluorescence intensities were recorded for several minutes in control buffer allowing the system to equilibrate. All detected parameters 10 minutes prior to the change of the perfusion buffer (i.e. control conditions) where averaged and set as 100%. During experiments, the cells were perfused using a home-built perfusion chamber to guarantee a continuous and noise-free exchange of the respective perfusion buffer and a physiological shear stress [20].

### AFM Imaging and Profile Analysis

The AFM is an ideal method to visualize cell surface, height and volume [30], [23]. To generate 3D topography maps, subconfluent GM7373 (∼90%) were scanned with cantilever (MSCT, Bruker). The scan size was set to 100 µm × 100 µm (256×256 pixels) with a scan rate of 0.375 Hz. These settings guarantee an almost noise-free measurement of cell height without disturbance of the cell topography by the indentation force [31].

### Analysis of eNOS Activity

eNOS activity was measured by analysis of DAF-FM DA (Merck, Darmstadt, Germany) fluorescence intensity, a specific fluorescent probe for NO [32], [8]. The interaction of actin and eNOS was inhibited using the recently introduced peptide P326TAT (Metabion, Martinsried, Germany) [11]. The inhibitor peptide consist of the 8 amino acid (aa) sequence of the eNOS binding site for actin (P326) tagged to the 10 aa TAT sequence (derived from the transduction domain of HIV TAT) which facilitates the uptake of this peptide into the cells. Thus P326TAT acts as an antagonist for the eNOS/actin interaction. For these experiments, GM7373 cells were seeded on 96-well plates (Becton Dickinson Labware, France) and cultured for 48 h until they reached confluence. Then, culture medium was replaced by the corresponding DAF-FM DA solution (final 10 µM) containing control buffer, 12 mM K^+^ buffer or 50 nM CD buffer each supplemented with a control peptide or P326TAT, respectively. The plate was incubated at room temperature on a shaker (240 rpm) for 3 h. Incubation was followed by a fluorescence read out using a Fluoroskan II plate reader (Thermo Labsystems). Fluorescence intensities of control values (control buffer + control peptide) were averaged and set to 100%. All other values were analysed relative to control conditions.

### Statistics

All correlations of stiffness and Lifeact-eGFP fluorescence intensity were performed in paired experiments. The obtained data were analysed using paired t-test if the values were parametrically distributed. If the results were non-parametric, they were analysed by Wilcoxon test. Data in all experiments are given as mean values ± standard error of the mean (SEM) of *n* experiments. NO detection was performed in unpaired experiments and analysed by ANOVA (+ Bonferroni test) due to parametric distribution of acquired data. Values are given as mean ± SEM with *N* numbers of used 96-well plates and *n* number of analysed wells. Results of all experiments were considered statistically significant if p<0.05 (indicated by *).

## Results

### Destabilization of the Cortical Cytoskeleton by Low Doses of Cytochalasin D

**Figure 3:**
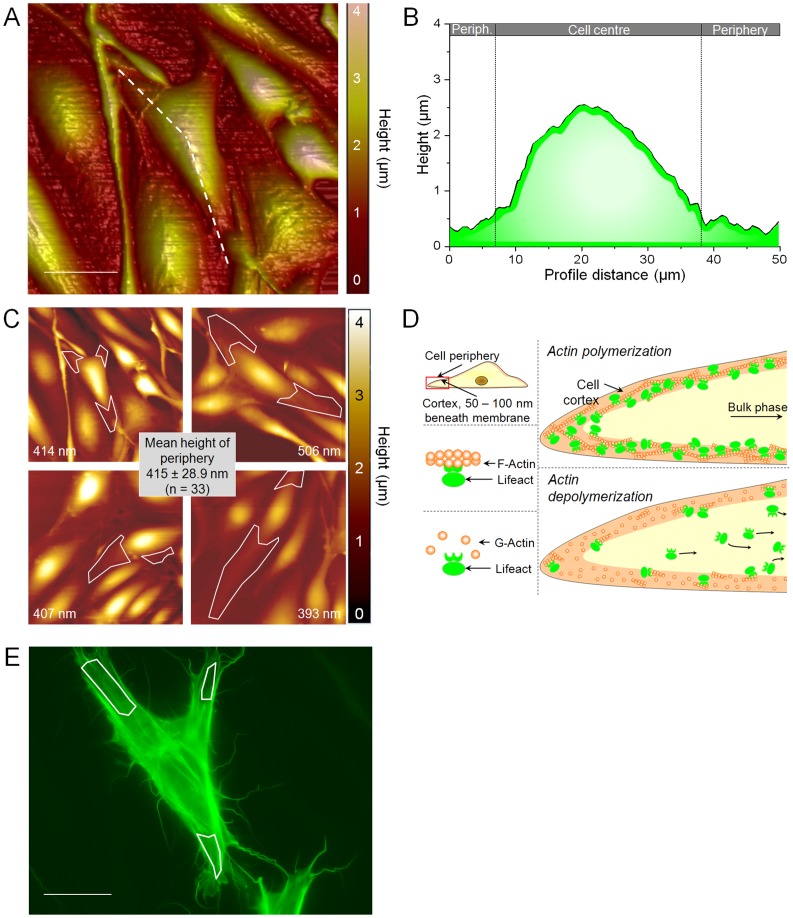
Cortical fluorescence in the cell periphery. a) 3D topography map of a confluent endothelial monolayer generated by an AFM surface scan. Cell height is illustrated by the colour code, shown at the right. The dashed line indicates the section for the height profile (scale bar = 20 µm). b) Height profile of the transfected endothelial cell (see also a). c) Height analysis of cell periphery. Four representative surface scans of endothelial cells are shown. Values indicate mean heights of the respective cell periphery. Quantification of all analysed cells (n = 33) is displayed. d) Proposed model of Lifeact-eGFP binding to actin filaments. Actin depolymerisation of cortical filaments leads to a dissociation of actin and Lifeact which results in a diffusion of fluorophores into the cell centre and a decrease of peripheral fluorescence intensity. e) Epifluorescence of the Lifeact-eGFP transfected cell (see also e). The white frames indicate the region of interest used for analysis of peripheral fluorescence intensity.

**Figure 4:**
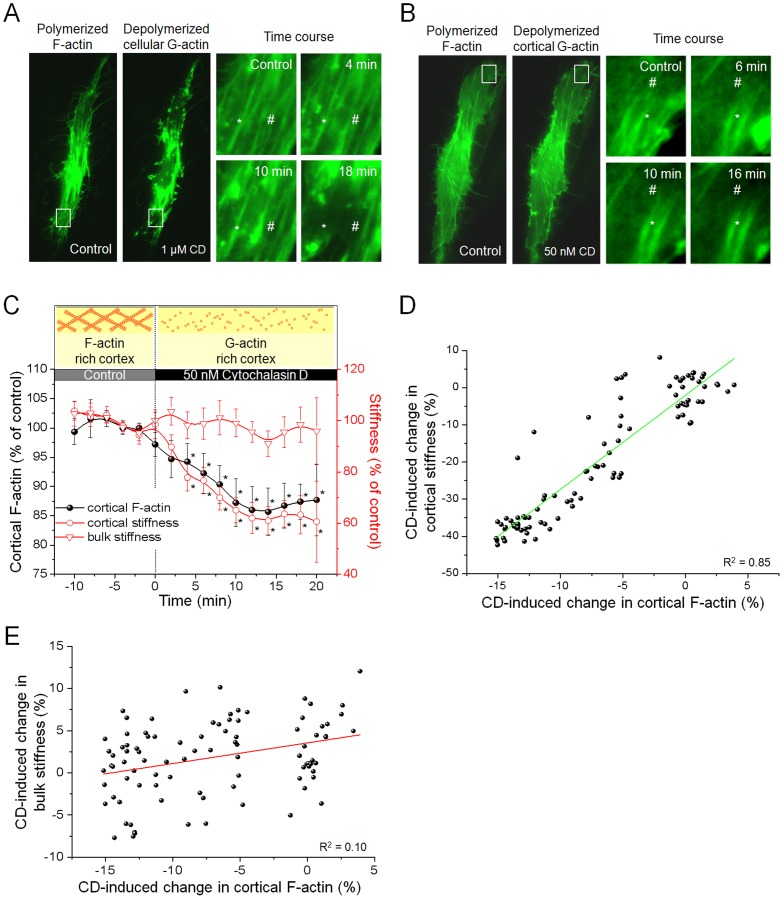
Proof of principle. a) Fluorescence images of a Lifeact-eGFP transfected cell (grown in a confluent monolayer of non-transfected cells) before and after treatment with 1 µM CD. Cellular as well as cortical F-actin depolymerizes upon treatment with CD. Highlighted details illustrate the time course of the CD effect. Cortical F-actin (#) as well as F-actin stress fibres (*) are destabilized. b) Lifeact-eGFP transfected cell (grown in a confluent monolayer of untransfected cells) treated with 50 nM CD. Application of low doses of CD destabilizes cortical F-actin (#) and spares bulk stress fibres (*). c) Simultaneous analysis of cortical F-actin ( = peripheral Lifeact-eGFP fluorescence intensity, black circles), cortical stiffness (К_cortex_, red circles) and bulk stiffness (К_bulk_, red triangles). Application of 50 nM cytochalasin D (CD) decreases cortical F-actin and К_cortex_ significantly (after 4 minutes, p<0.05) whereas К_bulk_ is unaffected (n = 7). d) Correlation of CD-induced changes of К_cortex_ and cortical F-actin. The correlation coefficient, R^2^ = 0.85, indicates a linear correlation. e) Analysis of the correlation between К_bulk_ and cortical F-actin indicates no correlation (R^2^ = 0.1).

Analysis of cortical fluorescence intensity by whole-cell epifluorescence microscopy faces the problem that bulk fluorescence, emitted by fluorophores from the cell interior, is hardly distinguishable from the fluorescence of cortically located fluorophores. This impedes the analysis of cortical actin using fluorescent probes and epifluorescence microscopy. On the other hand, high resolution microscopy techniques that might resolve the apical cell cortex (e.g. TIRF) cannot be matched with AFM-based stiffness measurements. However, the natural cell shape might allow measurements of cortical fluorescence by quantitative fluorescence analysis in the peripheral zones of the cell. The cell periphery is rather flat and thus dominated by the cell cortex. Hence, peripheral fluorescence is expected to be significantly determined by cortical fluorophores. The peripheral morphology can be investigated by AFM topography mapping. To do so, we performed AFM surface scans (Fig. 3a) of confluent monolayer of bovine aortic endothelial cells (GM7373). The analysis of the height profile of the cells (Fig. 3a, dashed line) demonstrates that the cells are, as expected, much flatter in the region of the cell periphery than in the cell centre (Fig 3b). Quantification of peripheral cell heights indicates an average height of 415±28 nm (n = 33). Figure 3c displays 4 representative surface scans (100×100 µm) of endothelial cells (peripheral regions marked by white frames). Due to the rather low height of the cell periphery, the fluorescence emitted by cortical (Lifeact-)fluorophores holds a higher fraction of the total intensity compared to the bulk fluorescence in the respective “far-away” regions. Hence, in a Lifeact-eGFP expressing cell, any depolymerisation of cortical F-actin induces a diffusion of the freed Lifeact fluorophores from the cortex into the bulk of the cell, resulting in a decrease of peripheral fluorescence intensity. In contrast, any polymerization may recruit new Lifeact molecules, which leads to an increase of peripheral intensity (Fig. 3d). This enables the investigation of dynamic processes in the cell cortex of living endothelium by epifluorescence microscopy. We analysed the fluorescence intensity in the peripheral regions of Lifeact-eGFP transfected cells to detect changes in cortical F-actin, as depicted in figure 3e (circled regions, same cell as in Fig. 3a, 3c). As a proof of principle that cortical actin dynamics determines cortical stiffness, we destabilized the cortical cytoskeleton with cytochalasin D (CD). Large doses of CD, commonly used to destabilize the actin cytoskeleton (∼ 1 µM CD), induce a non-selective depolymerisation F-actin [13], [33]. As shown in figure 4a, whole cell F-actin of a Lifeact-eGFP transfected cell (sitting in a non-transfected - and thus non-fluorescent - monolayer) is found depolymerized 18 minutes after CD application. The close-up views of the cell periphery illustrate that cortical F-actin (indicated by #) as well as actin stress fibres (*) are destabilized. However, as shown in figure 4b, application of a 20-times low dose of CD (50 nM) selectively destabilizes cortical F-actin (#) while classical stress fibres (*) are unaffected. In average, peripheral Lifeact fluorescence, and thereby cortical F-actin, was reduced to 86.7±3.8% of control after 16 minutes of CD treatment (Fig. 4c, black circles). The selective destabilization of the cortical actin can be simultaneously analysed by the AFM-based nanoindentation technique [29], [34]. This technique allows performing one-dimensional stiffness tomography of living cells using force-indentation curves (F/I-curve, for details see methods part). Analysis of the slope of these F/I-curves allows a quantification of stiffness in different layers (compartments) of the cell. Both parameters, cortical and bulk stiffness, of living endothelial cells are constant under control conditions (Fig. 4c, n = 7). The minor variations of up to 2% are due physiological oscillations of К_cortex_ induced by actin dynamics [35]. Immediately after applying 50 nM CD, К_cortex_ decreases (Fig. 4c, red circles). The effect is significant after 4 minutes (p<0.05) and reaches a new plateau after approx. 16 minutes at 63.6±5.10% of control (1.9±0.16 pN/nm to 1.2±0.11 pN/nm). In contrast to the cortex, the К_bulk_ is not affected by these low concentrations of CD and still exhibits a stiffness of 98.7±6.0% of control (19.1±1.12 pN/nm to 18.9±1.07 pN/nm, Fig. 4c, red triangle). Hence it is possible to perform an optical and nanomechanical sectioning of the cell while selectively influencing the thin cell cortex. These results indicate that К_cortex_ is an expression of cortical actin dynamics. The described decrease in fluorescence intensity correlates directly with the softening of the cell cortex, since changes in К_cortex_ and cortical F-actin show an almost linear dependence (R^2^ = 0.85, Fig. 4d). Modifications in К_bulk_ and cortical F-actin, however, show no correlation at all (R^2^ = 0.12, Fig. 4e).

### Physiological Depolymerisation of Cortical F-actin

**Figure 5:**
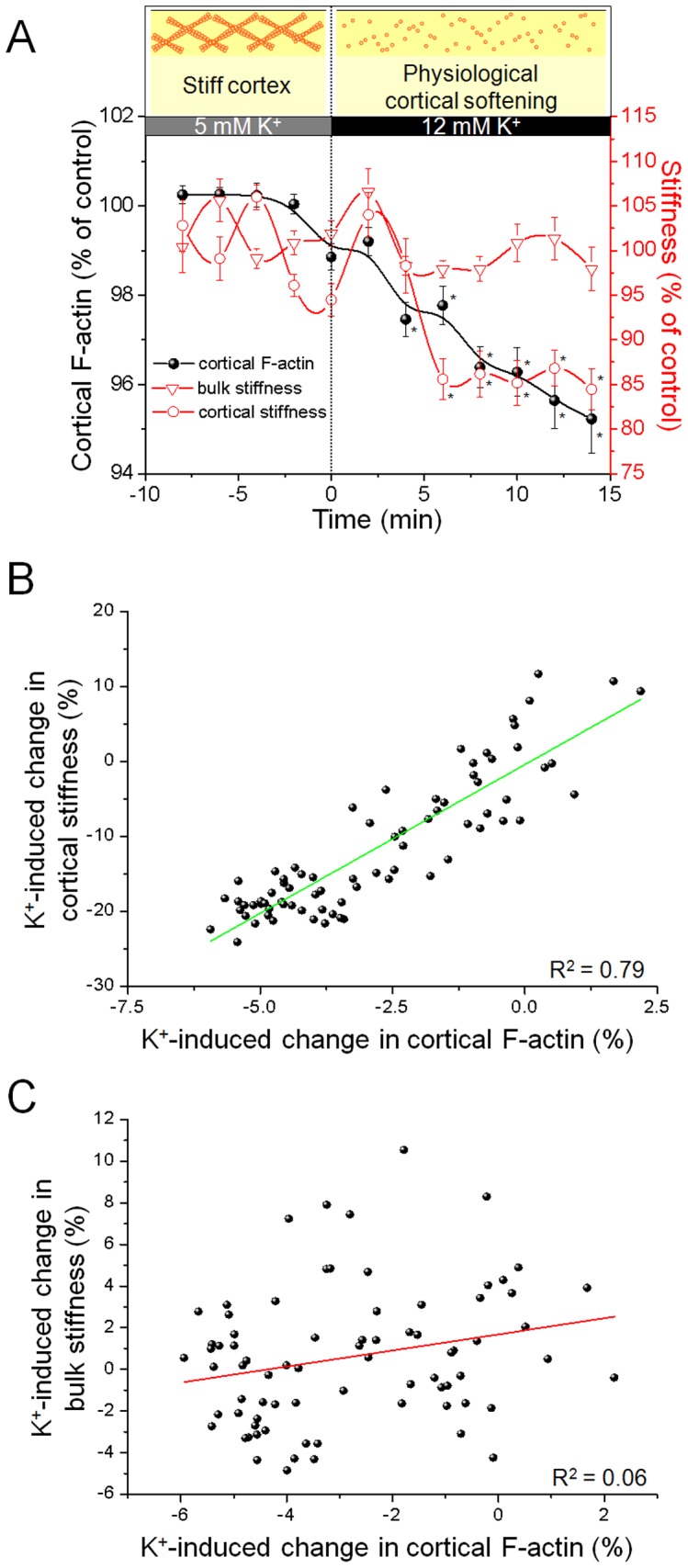
Physiological relevance. a) Simultaneous measurement of cortical F-actin intensity (black circles), cortical stiffness (К_cortex_, red circles) and bulk stiffness (К_bulk_, red triangles). After an increase of [K^+^]_e_ from 5 to 12 mM cortical F-actin and К_cortex_ decrease. The effect is significant after 6 minutes (n = 12). К_bulk_ is not affected by an increased [K^+^]_e_. b) Changes in К_cortex_ and Lifeact-eGFP intensity show an almost linear correlation (R^2^ = 0.79), c) whereas the correlation of K^+^-induced changes in cortical F-actin and К_bulk_ do not correlate (R^2^ = 0.06).

Although CD is a useful agent to depolymerise actin, it is a rather unphysiological stimulus. To verify that actin dynamics is a major determinant of К_cortex_, we induced a change in К_cortex_ by varying the external potassium concentration within a physiological range. It is known that, in the healthy human organism, physiological plasma potassium concentrations vary around 3.7–5.3 mM. However, locally, extracellular potassium ([K^+^]_e_) can rise up to 12 mM due to increased muscle or nerve cell activity [36], [37]. Previous data from our lab demonstrated that high [K^+^]_e_ induces a significant softening of the cortex paralleled by an increase of NO bioavailability [5]. Hence, if cortical actin dynamics is indeed a functional component of К_cortex_, an increase in [K^+^]_e_ should depolymerize cortical actin and thereby decrease К_cortex._ Therefore, we analysed the effect of high [K^+^]_e_ on К_cortex_ and cortical actin dynamics. As shown in figure 5a, both parameters, К_cortex_ (red circles) and cortical F-actin concentration (black circles), show an immediate decrease as soon as [K^+^]_e_ is isoosmotically increased from 5 to 12 mM, which is significant after 6 minutes (n = 12, p<0.05). After 14 minutes К_cortex_ reaches a new plateau at 84.4±2.3% of control (1.6±0.06 pN/nm to 1.4±0.05 pN/nm). Simultaneously cortical F-actin decreases to 95.2±0.7% of control. The correlation coefficient of R^2^ = 0.79 indicates an almost linear response (Fig. 5b). In contrast, К_bulk_ is not affected by [K^+^]_e_ as is remains constant and shows a stiffness of 97.9±2.40% of control (19.1±0.84 pN/nm to 18.6±0.80 pN/nm). Similar to the CD-dependent effect, there is, due to a correlation coefficient of R^2^ = 0.06, no correlation of cortical F-actin and К_bulk_ (Fig. 5c). Taken together, a physiological trigger, e.g. [K^+^]_e_, depolymerizes the submembranous actin web and thus explains the decrease of К_cortex_.

### Lifeact Fluorescence and Cell Volume

**Figure 6:**
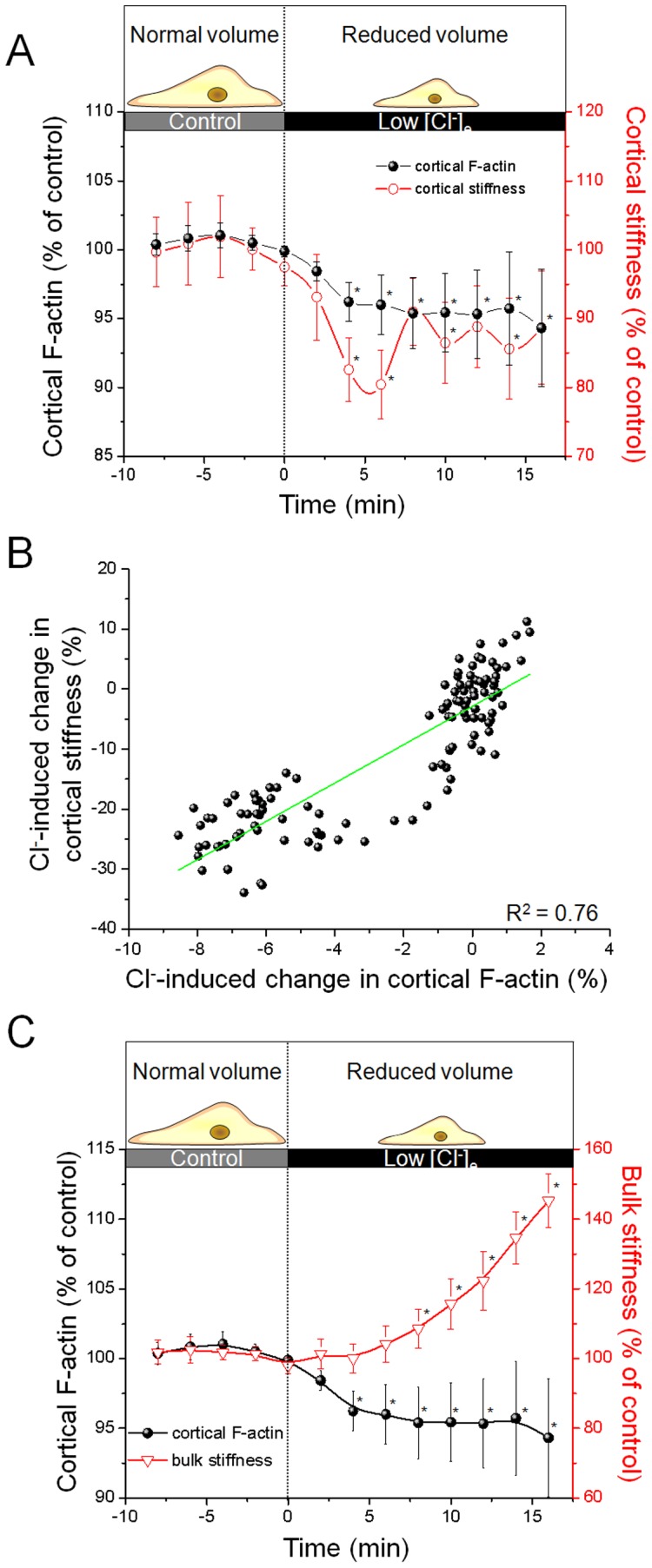
Exclusion of volume artefacts. a) Effect of low [Cl^-^]_e_ on cortical F-actin (black circles) and К_cortex_ (red circles) of endothelial cells are detected simultaneously. Changing to low [Cl^-^]_e_ decreases stiffness and F-actin in the cell cortex. b) Correlation of low [Cl^-^]_e_-induced changes between cortical F-actin and К_cortex_. The correlation coefficient of R^2^ = 0.76 indicates a linear dependence (n = 8). c) Effect of low [Cl^-^]_e_ on К_bulk_ (triangles) and cortical F-actin (black circles). While cortical F-actin depolymerizes (see also d), К_bulk_ increases simultaneously.

The described method to analyze cortical actin dynamics based on measurements of fluorescence intensity in the cell periphery. However, it is known that CD and [K^+^]_e_ influence cell volume [5] and cell volume regulation [38]. Therefore, it cannot be excluded, that the described effects on cortical Lifeact-eGFP fluorescence are due to a swelling-induced dilution of fluorophores in the analysed regions and not, as postulated, to an expression of F-actin dynamics. A swelling-induced dilution of fluorophores may lead to diminished fluorescence intensity and thereby to a misinterpretation regarding the cytoskeletal organization. To eliminate this possibility, we treated the transfected endothelial cells with low extracellular chloride ([Cl^-^]_e_) and simultaneously analysed К_cortex_ and Lifeact-eGFP fluorescence intensity. An isoosmotic decrease in [Cl^-^]_e_ reduces cell stiffness but is, in contrast to the effect of high [K^+^]_e_, accompanied by a significant decrease in cell volume [31]. If the effect of high [K^+^]_e_ on cortical Lifeact-eGFP fluorescence was only due to a dilution of fluorophores, based on an increase of cell volume, a Cl^–^induced decrease in cell volume should exert the opposite effect. Accordingly, low [Cl^-^]_e_ should increase fluorescence intensity because of cell shrinkage despite cortical softening. However, as shown in figure 6, this is not the case. A change to low [Cl^-^]_e_ conditions induces a decrease in cortical Lifeact-eGFP fluorescence intensity (n = 8). After 10 minutes, Lifeact-eGFP intensity (black circles) and К_cortex_ (red circles) decline to 95.4±2.8% and 86.5±5.8% (1.8±0.10 pN/nm to 1.6±0.08 pN/nm), respectively (Fig. 6a). The effect of low [Cl^-^]_e_ is significant after 4 minutes (p<0.05) and shows an almost linear correlation with a correlation coefficient of R^2^ = 0.76 (Fig. 6b). Interestingly, the induced cell shrinkage significantly affected К_bulk_ as it increases after application of low [Cl^-^]_e_ conditions (Fig. 6c). This effect may be due to the higher concentration of cell mass (i.e. proteins, organelles, vesicles etc.) in the cytoplasm. Taken together, these findings indicate that the observed correlation of cortical stiffness and Lifeact-eGFP intensity cannot be explained by changes in cell volume. Hence cortical Lifeact-eGFP intensity indeed represents submembranous F-actin dynamics. Furthermore, the simultaneous analysis of К_cortex_ and К_bulk_ underlines the independent role of the cell cortex as a functional compartment within the cell.

### Actin-associated К_cortex_ and eNOS/actin Interaction

**Figure 7:**
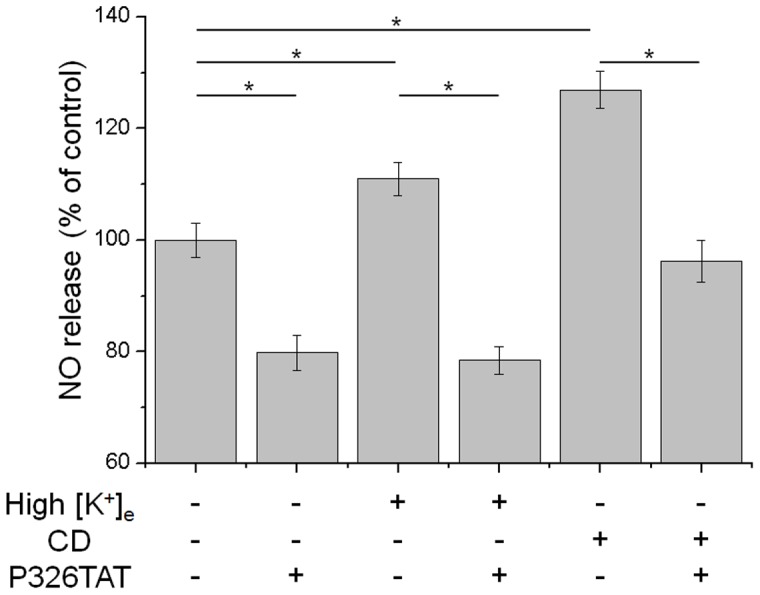
eNOS actin interaction. Effect of high [K^+^]_e_ and CD on endothelial NO release. Cells loaded with the fluorescent NO indicator DAF-FM DA were incubated in control buffer, high [K^+^]_e_ or CD in the presence of control peptide or P326TAT, respectively (N = 5, n = 30). DAF-FM fluorescence intensity under control conditions was averaged and set to 100%. Respective values are given as % of control ± SEM. High [K^+^]_e_ and CD significantly increase NO release relative to control. In all approaches P326TAT inhibits the eNOS/actin interaction and thereby the NO release.

After verifying that cortical actin dynamics is a major determinant of cortical stiffness, we addressed the question whether a decrease in cortical F-actin, and thus a larger G-actin content, also causes increased eNOS activity. It has been shown before that eNOS activity is stimulated by G-actin [11]. Hence, elevation in cortical G-actin should lead to increased G-actin/eNOS association and thereby to NO release. To confirm this presumption, we analysed the effect of [K^+^]_e_ and CD on endothelial NO release, with and without inhibition of the eNOS/actin interaction. eNOS activity was measured using DAF-FM DA, a fluorescent probe which detects intracellular released NO [32], [8]. To inhibit the eNOS/actin interaction, P326TAT was applied to the cells. The 18 amino acid peptide P326TAT exhibits an eNOS-homologue binding domain for actin which “masks” actin and thereby inhibits eNOS/actin association [11]. After loading the cells with DAF-FM DA, eNOS activity was analysed in the presence of P326TAT or a control peptide (N = 5, n = 30). eNOS activity under control conditions was averaged and set to 100%. As shown in figure 7, P326TAT significantly decreases eNOS activity to 79.8±3.1% of control conditions. 12 mM [K^+^]_e_ as well as 50 nM CD significantly increases NO production to 111.0±2.9% and 127.0±3.4% of control (p<0.05). In both cases, endothelial NO release is significantly decreased when the eNOS/actin interaction is inhibited by P326TAT. The inhibiting peptide reduced eNOS activity to 78.4±2.5% of control under high [K^+^]_e_. CD-stimulated eNOS activity is lowered by P326TAT to 96.2±3.8% of control (Fig. 7). The data indicate that cortical NO release is indeed mediated by a depolymerisation of F-actin in the cell cortex.

## Discussion

**Figure 8:**
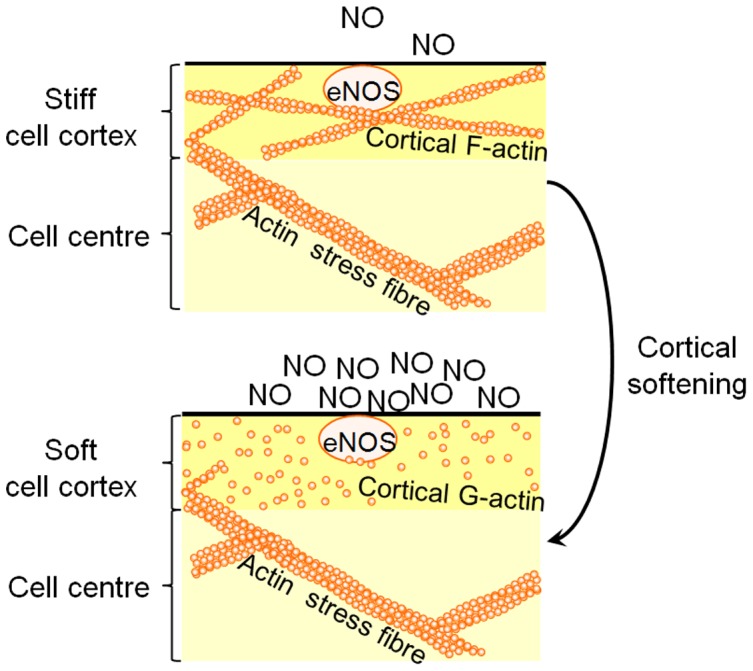
Proposed model of cortical stiffness-mediated eNOS activity. A stiff cortex implies a high rate of actin polymerization. eNOS, associated to actin filaments, exhibits a low activity. Actin depolymerisation causes cortical softening. Free G-actin, instead of F-actin, binds to eNOS stimulating its activity.

Over the last few years, accumulating evidence highlighted the importance of the cell cortex as a key structure of the cell. The endothelial cell cortex, and especially the cortical actin web, is a highly dynamic structure which determines vascular physiology [33], [39], [40]. Additionally, the cell cortex seems to influence ubiquitous, and thus not endothelium-specific, signalling mechanisms involved in cell cycle, cell migration and cell aging [16], [41], [42], [15]. This is an intriguing finding, considering the small fraction of the cell cortex compared to total cell volume. It is comparable with the thickness and volume of a chicken egg shell, which performs a crucial function for the eggs “survival”, relative to the total egg volume. Endothelial cells have an average volume of about 1700 fl and an apical surface area of 1200 µm^2^ [43]. For the apical cell cortex, with a height of about 75 nm [7], a volume of about 90 fl is calculated. Thereby the contribution of the apical cell cortex to total volume exhibits only about 5%. Despite its small size, the cell cortex appears to exhibit extraordinary physiological relevance partly due to its variable nanomechanical properties. As shown here, endothelial cells can respond to certain stimuli by de−/polymerization of cortical actin which results in fast modulation of eNOS activity. This view supports the hypothesis that the cell cortex switches from gel to sol and thereby modulates its function [5]. As summarized in figure 8, a stiff gel-like cortex consists of mainly F-actin associated to eNOS. Subsequently, eNOS activity is reduced which triggers vasoconstriction. Cortical solation, however, is due to a decrease in the F-actin/G-actin ratio. Association of G-actin with eNOS results in elevated NO release and thus induces dilation of vascular smooth muscle cells. This actin polymerization dependent and thus stiffness dependent eNOS activity adds a new piece of puzzle to the understanding of the stiff endothelial cell syndrome [9]. In this syndrome, the increased stiffness of the endothelial cell is postulated to cause endothelial dysfunction. Our findings show that cortical nanomechanics reflects the dynamics in the submembranous actin web.

Additionally, actin-dynamics dependent eNOS regulation might represent a part of the physiological function of the vasculature. From human lung smooth muscle cells it is known that cells can respond to stretch with a solation of the cytoskeleton and a subsequent softening of the cell [44]. It is likely that cortical solation also occurs rhythmically in vascular endothelial cells in vivo, when shear forces induced by pulsatile blood flow act on the endothelial surface. The proposed solation increases the G-actin/eNOS interaction and thereby regulates vessel diameter. A decrease of blood pressure (by any reason) results in a reduced shear stress which causes a gelation (cortical actin polymerization) suppressing eNOS activity followed by vasoconstriction [44]. The balance between gelation and solation could adjust tissue perfusion to the demand of cell metabolism.

It should be mentioned that other factors than actin de/polymerization may well contribute to cortical stiffness. Water content and amount of dry mass (i.e. proteins, vesicles and organelles) as well as motor proteins (e.g. myosin, kinesin) play an important role in the determination of cellular nanomechanics [10], [45], [16], [35], [46]. Furthermore, the fact that the increase in eNOS activity induced by cytochalasin D cannot be inhibited completely by P326TAT (the inhibitor of eNOS/actin interaction), indicates that other signalling pathways may contribute to stiffness-dependent eNOS activity. Nevertheless, their role in stiffness-regulated eNOS activity appears to be probably minor, since eNOS interacts with G- and F-actin and so far any interaction of eNOS and e.g. myosin has not been described yet [11]. However, it is still unclear how de−/polymerization of cortical actin is mediated. One hypothesis is that the intracellular ion concentration *per se* influences the actin polymerization [47]. Additionally, it is likely that, besides NO metabolism, other signalling mechanisms are affected by changes in cortical stiffness.

## Supporting Information

Figure S1 **Topography of the endothelial cell surface.** a) Confluent GM7373 endothelial cells were imaged by AFM. The colour scale indicates the cell height. b) Magnifications of the two regions marked in part a). Cell surface appears to be rather smooth. Occasionally, filaments of the cortical actin cytoskeleton (arrowheads) as well as small invaginations (arrows) can be detected on the cell surface.
(TIF)
