# Supplementary figures and images for: Cortical Actin Nanodynamics Determines Nitric Oxide Release in Vascular Endothelium
Source: PLoS One. 2012 Jul 23;7(7):e41520. doi: 10.1371/journal.pone.0041520 (PMC3402397; doi:10.1371/journal.pone.0041520)

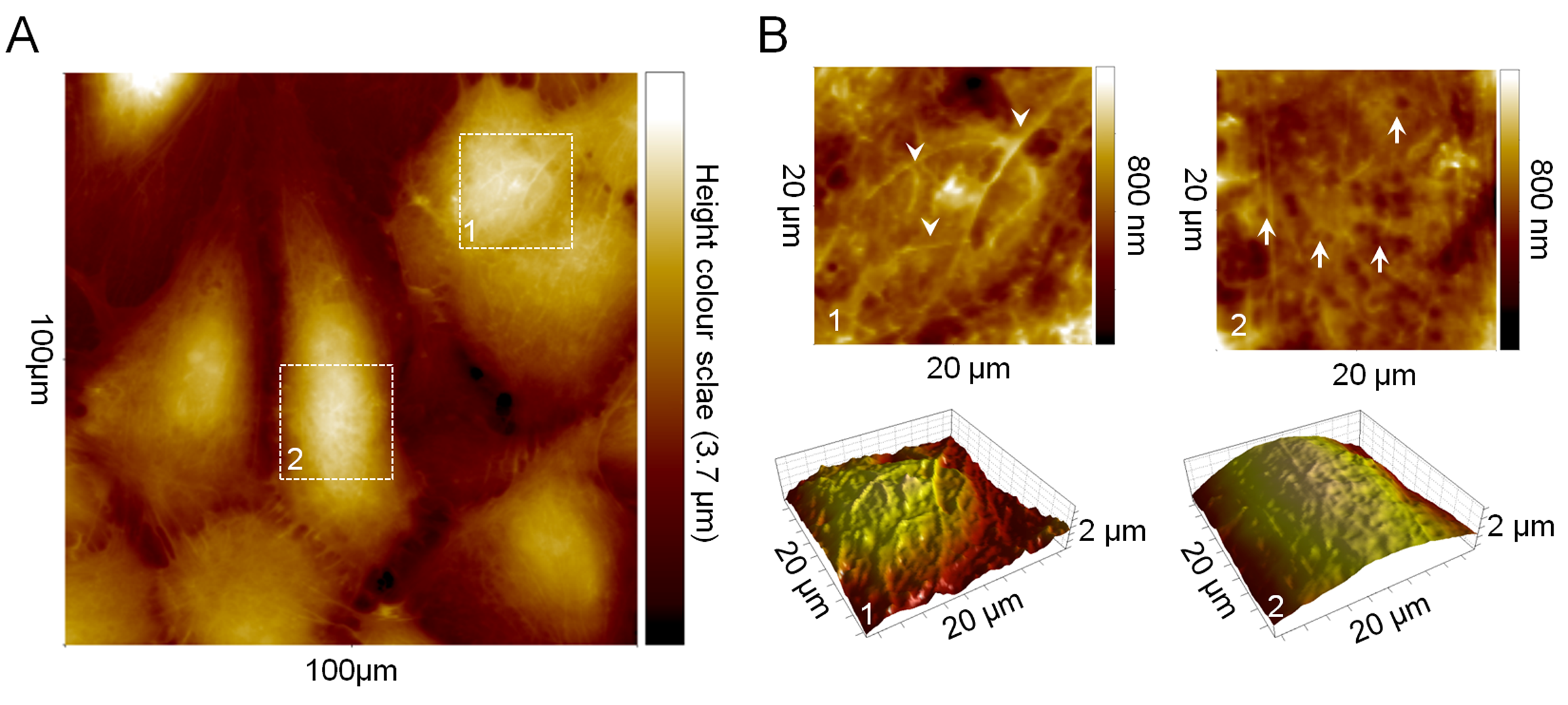

Supplement: Figure S1 — Topography of the endothelial cell surface. a) Confluent GM7373 endothelial cells were imaged by AFM. The colour scale indicates the cell height. b) Magnifications of the two regions marked in part a). Cell surface appears to be rather smooth. Occasionally, filaments of the cortical actin cytoskeleton (arrowheads) as well as small invaginations (arrows) can be detected on the cell surface. (TIF) [file pone.0041520.s001.tif]
